# Behavioural Determinants of Hand Washing and Glove Recontamination before Aseptic Procedures at Birth: A Time-and-Motion Study and Survey in Zanzibar Labour Wards

**DOI:** 10.3390/ijerph17041438

**Published:** 2020-02-24

**Authors:** Giorgia Gon, Sandra Virgo, Mícheál de Barra, Said M. Ali, Oona M. Campbell, Wendy J. Graham, Stephen Nash, Susannah L. Woodd, Marijn de Bruin

**Affiliations:** 1 London School of Hygiene and Tropical Medicine, Faculty of Epidemiology and Population Health, London WC1E 7HT, UK; oona.campbell@lshtm.ac.uk (O.M.C.); wendy.graham@lshtm.ac.uk (W.J.G.); Stephen.nash@lshtm.ac.uk (S.N.); Susannah.woodd@lshtm.ac.uk (S.L.W.); 2 University of Kent, Higher Education Access Tracker, Catenrbury CT2 7NZ, UK; s.virgo@kent.ac.uk; 3 Department of Life Sciences, Brunel University London, Uxbridge UB8 3PH, UK; Micheal.DeBarra@brunel.ac.uk; 4 Public Health Laboratory-Ivo de Carneri, Chake Chake, Pemba, Zanzibar 9820, Tanzania; saidmali2003@yahoo.com; 5 Institute of Applied Health Sciences, University of Aberdeen, Aberdeen AB24 3FX, UK; Marijn.deBruin@radboudumc.nl; 6 Radboud University Medical Center, Radboud Institute for Health Sciences, IQ healthcare, 6525 GA Nijmegen, The Netherlands

**Keywords:** hand hygiene, determinants, birth

## Abstract

Recent research calls for distinguishing whether the failure to comply with World Health Organisation hand hygiene guidelines is driven by omitting to rub/wash hands, or subsequently recontamination of clean hands or gloves prior to a procedure. This study examined the determinants of these two behaviours. Across the 10 highest-volume labour wards in Zanzibar, we observed 103 birth attendants across 779 hand hygiene opportunities before aseptic procedures (time-and-motion methods). They were then interviewed using a structured cross-sectional survey. We used mixed-effect multivariable logistic regressions to investigate the independent association of candidate determinants with hand rubbing/washing and avoiding glove recontamination. After controlling for confounders, we found that availability of single-use material to dry hands (OR:2.9; CI:1.58–5.14), a higher workload (OR:29.4; CI:12.9–67.0), more knowledge about hand hygiene (OR:1.89; CI:1.02–3.49), and an environment with more reminders from colleagues (OR:1.20; CI:0.98–1.46) were associated with more hand rubbing/washing. Only the length of time elapsed since donning gloves (OR:4.5; CI:2.5–8.0) was associated with avoiding glove recontamination. We identified multiple determinants of hand washing/rubbing. Only time elapsed since washing/rubbing was reliably associated with avoiding glove recontamination. In this setting, these two behaviours require different interventions. Future studies should measure them separately.

## 1. Introduction

Hand hygiene of birth attendants is a key infection prevention act for both mothers and newborns worldwide [1,2,3]. Indeed, hand hygiene is considered the single most important intervention to reduce healthcare-associated infections (HAIs) [4]. These infections affect 15% of patients in low and middle-income countries (LMICs) [5], including Tanzania which is our study context [6]—twice as high than in Europe [7]. In low-resource settings, newborns born in hospital are 3–20 times more likely to develop an infection compared to their European counterparts [8]; one study suggests that 4% of mothers contract puerperal sepsis in Tanzania [2]. Together with rapidly-growing numbers of women delivering in healthcare facilities in Tanzania [9], overcrowding and unpredictable staffing levels and resources are frequent [10], and the need for adequate infection prevention is paramount.

Inadequate hand hygiene (HH) compliance amongst healthcare personnel is common [11,12] and is usually summarized as a single behaviour. However, in our previous work in Zanzibar (Tanzania), we identified the need to distinguish whether the failure to comply with the hand hygiene guidelines stemmed from omitting to rub/wash hands, or the process of subsequently avoiding recontamination of hands/gloves before a procedure [13]. This distinction cannot be made using the current WHO HH Observation Form [14]; yet, because these different behaviours may have different determinants, it is potentially important to study them separately in order to develop optimally effective interventions.

Previous studies have stressed the importance of investigating both the contextual and individual determinants of healthcare workers’ hand hygiene [12,15]. The contextual factors include workload [11,12,16,17], staff professional background [11,12,15], and availability of necessary materials such as soap and water [11,12]. The individual factors include constructs like knowledge [12,18], and healthcare workers’ attitudinal [12,15,17,19], normative [12,15,17,20], and control beliefs [12,21]. Various social cognitive theories include these individual factors, although context is usually described in very general terms (e.g., barriers and facilitators). In this study, we employ the widely-used Integrated Behavioural Model (IBM) [22] as the organizing framework, because it integrates individual and contextual behavioural determinants from multiple theories in one comprehensive model. The importance of using behavioural theory to guide research and implementation in this area has been highlighted [12,23,24].

To our knowledge, no prior studies have quantitatively examined the determinants of recontamination. Therefore, our main objective was to investigate the independent association between individual and contextual determinants with hand rubbing/washing, and separately with avoiding glove recontamination (preceded or not by hand rubbing/washing); as well as compare these. We focused on determinants that were likely to be modifiable.

## 2. Materials and Methods

HANDS was a mixed-methods study that ran between November 2015 and April 2017 in the 10 highest-volume labour wards in Zanzibar (which we selected according to the reported volume across all the 37 facilities providing maternity services), with average monthly deliveries of 75 to 930. The project was a partnership between the University of Aberdeen, The London School of Hygiene and Tropical Medicine, and the Public Health Laboratory-Ivo De Carneri. Previous work in eight of these maternity wards found the majority had policies and basic material and infrastructure to perform HH but only 50% had received HH training in the previous year [25].

### 2.1. Study Design and Instruments

Within HANDS, between September and December 2016, we used time-and-motion methods and a cross-sectional survey to capture the HH behaviour and its determinants amongst 103 birth attendants. We used the STROBE guidelines to design and report this study as described in Gon et al. [13]. For the time-and-motion component, three observers (trained midwives) used an observation tool to record hand actions (e.g., procedures, hand touches on surfaces) of birth attendants 24 h per day, for a mode of 6 days (range: 5–14 days) in each of the 10 labour wards. They also collected information on the availability of key materials for hand hygiene (e.g., water) and on the presence of the ward in-charge during each observation session. Data were collected via tablets, and the observation tool was pre-coded using WOMBATv2 software [26,27]. More details on the use of this tool including piloting, training, data cleaning are described in Gon et al. 2018 [13]. We calculated several sample size scenarios for a cross-sectional design using EpiInfo v7 by varying the ratio of unexposed to exposed, the percentage of outcome in the unexposed group, and the effect size. For example, we had 80% power to capture an effect size of 2 (or above) when the distribution of the outcome in the unexposed was 10% (or above) and the ratio of unexposed to exposed ratio was 5:1 (Supplementary A).

For the survey, the same data collectors administered a questionnaire, lasting about 45 min, to all birth attendants observed at each facility. Generally, the questionnaire was administered shortly (1–19 days) after observation in each facility, with one exception, where it was three months later. The questionnaire was administered after the observation in order to conceal the specific study objectives from the birth attendants during the observation period. To further reduce the risk that the observational study biased survey responses [16], we aimed for a birth attendant to not be interviewed by the data collector who observed them. For 7/103 birth attendants, this was not possible because all three data collectors had observed the participant.

The questionnaire (available in Supplementary B) included questions on the socio-demographic characteristics of the respondents and psychological constructs stipulated by the Integrated Behavioural model [22]. The psychological constructs were asked for two outcomes separately (hand rubbing/washing and avoiding glove recontamination) specifically for the scenario of preparing for a delivery, which is a key infection prevention moment. Questionnaire items were also informed by the findings of earlier qualitative work within HANDS (manuscript under preparation), via a literature review, drawing on existing questionnaires and approaches as detailed below [19,22,28,29,30]. The questionnaire was administered in Swahili.

The questionnaire was piloted twice, respectively administering it to three and nine birth attendants, and revised accordingly. Pilot testing suggested a two-stage approach for eliciting responses about the psychological constructs (e.g., 1. Do you agree or disagree? 2. Do you agree/disagree a little or a lot?) understood best. Even though we tried to keep the number of items and responses options consistent across outcomes and constructs, the pilot results suggested some questions and response options did not work within our context (for example, answers were at ceiling). Therefore, the number of items or response options differ for different constructs in the final version of the questionnaire. The training for this tool was done over two days.

In developing the psychological constructs that we measured using multiple items with Likert-like responses, we excluded two items that were intended to be reverse-scored but whose eventual distribution indicated that they had not been understood that way (details in Table S3 of Supplementary C). We used Cronbach’s alpha to investigate reliability of the constructs. Individual items were removed if this increased Cronbach’s alpha by a substantial amount (details in Table S3 of Supplementary C). Due to item removal, the final scales have a variable number of items. Sets of items with low internal reliability (alpha < 0.6) were not used. These were, from Table 1, instrumental attitudes for both outcomes, and experiential attitudes for hand rubbing/washing. Items were combined to make a summative rating scale by calculating the mean score across all of them. Details of how we measured each construct and their internal reliability are available in Supplementary C. We describe in more detail the relevant questionnaire variables.

### 2.2. Variables and Their Operationalization

From the variables collected during observation and the questionnaire we selected, a priori, candidate modifiable determinants for hand rubbing/washing and for avoiding glove recontamination (listed in Table S1 of Supplementary C). A variable was subsequently excluded if (a) it did not have a sufficient distribution within the sample (e.g., availability of gloves); (b) it only related to a sub-group of HH opportunities for which the sample size was too small (i.e., whether the delivery equipment was collected or organized in a delivery set). Details of exclusion of separate variables can be found in Table S2 of Supplementary C. The following section focuses on the variables that we used for analysis in this paper.

#### 2.2.1. Variables Collected During Observation

##### Outcomes

We investigated determinants of two outcomes: whether before aseptic procedures during birth, birth attendants complied to hand rubbing/washing (outcome 1) and avoiding glove recontamination (outcome 2), preceded or not by hand rubbing/washing. In the latter, we included both opportunities where birth attendants did and did not hand rub/wash prior to donning gloves because the relevant set of individual determinants for this outcome collected with the questionnaire referred to avoiding glove recontamination in general. Compliance is achieved when hand rubbing/washing or avoiding recontamination after glove donning is performed when this should occur. The denominator is called a HH opportunity e.g., when the hand hygiene behaviour is expected to happen. These outcomes were operationalised using WHO guidelines [12,31,32] and are described in Gon et al. [13].

##### Contextual Modifiable Determinants

We constructed a proxy for workload, which was defined as the number of procedures conducted per minute in the interval between the start of the observation session and the opportunity of interest. Workload was categorised into five quintiles. The availability of single-use drying material was also collected during each observation session (categorised as binary). We also constructed a categorical variable measuring the time elapsed since donning gloves (less than a minute, between 1 and 2 min, between 2 and 3 min, and more than 3 min).

##### Candidate Confounders

For the relationship between each modifiable determinant and the outcome of interest, we drew a conceptual diagram, based on our reasoning and existing literature, to guide our selection of candidate confounders. From the observation tool we included: the presence of the in-charge (yes or no) even though the distribution of this variable in the sample was limited, and whether water and soap or handrub were available (which we refer to as necessary material).

#### 2.2.2. Variables Collected with the Questionnaire

##### Individual Modifiable Determinants

##### Training

A binary variable indicated whether the birth attendant received or not a refresher training that included the topic of hand hygiene in the last year.

##### Attitudes

People’s attitudes are comprised of beliefs about the outcomes or consequences of hand hygiene (instrumental attitudes), and experiential attitudes—the emotional or affective responses of healthcare workers when engaging in hand hygiene [22]. both have previously been found to be associated with hand hygiene [12,15,17,19]. The experiential attitudes scale for hand rubbing/washing was not used due to its low internal validity (Table 1). We measured experiential attitudes for avoiding recontamination with three items (questions) using a 3-point scale (no, yes a little, yes a lot). One item was removed to improve internal reliability. An example of an item asked was: “When you briefly touch the register, pen, or phone after putting on gloves, do you feel your hands are too dirty to conduct a delivery?” Experiential attitudes for avoiding recontamination did not show a continuous distribution. Hence, it was recoded into a binary variable indicating whether a respondent answered “yes a lot” to two items, in contrast to any other mixed response. Although we measured instrumental beliefs using Likert-like responses, this construct showed poor internal reliability for both outcomes and was not used (Table 1). Instead, we used a measure of instrumental beliefs, which relied on a scenario asking participants to list all possible causes of umbilical cord infection in a one-week old baby, noting any mention of hand hygiene (binary: mention HH, or not). The use of the scenario was tried during the qualitative phase of the project and worked well among participants.

##### Norms and Sanctioning

Norms and social influence can drive hand hygiene [12,15,17,33]. Norms were assessed using methods developed by Bicchieri et al. [29] and entailed asking the respondents about the number, out of 10, of (a) colleagues and (b) Zanzibar maternity managers they believed to be always hand rubbing/washing or avoiding recontamination before delivery—empirical expectations (referred by the IBM as descriptive norms). We also measured normative expectations (referred by the IBM as injunctive norms) in a similar way but responses were at ceiling and we could not use this construct.

Sanctioning is an important component of normative expectations [29]. During the qualitative phase of HANDS, we found that sanctioning in our context takes the form of gentle reminders. To measure the frequency of such reminders in facilities, we asked birth attendants whether, in the past month, they reminded anyone about hand rubbing/washing; whether they were reminded; whether they heard a manager reminding anyone; whether they heard a colleague reminding anyone. Each question was scored 0 (reminders absent) or 1 (present), and summed to create a variable with a range of 0–4.

##### Self-Efficacy

Self-efficacy is determined by one’s degree of confidence in the ability to perform the behaviour in the face of constraints and obstacles [22]. The broader umbrella of personal agency (which also includes perceived control) has been found to be associated with hand hygiene [12,21]. Self-efficacy was measured with four items for hand rubbing/washing and three items for avoiding recontamination respectively using a 3-point scale (very sure, a little sure, not sure). An example is: “How sure are you that you will (can) wash hands before every delivery when water is not flowing from the sink? Self-efficacy was rescaled to a 10-point distribution for easier interpretation.

##### Habit

Experience performing hand hygiene might make it habitual, an automatic response [34]. We measured habit with four items drawing from the proposed habit index by Gardner et al. [30]. Responses were measured on a 3-point scale (no, yes usually, yes always). One item was removed as it improved internal reliability of the scale. An example is: “Do you avoid touching unsterile objects before a delivery without thinking?” Habit was rescaled to a 10-point distribution for easier interpretation.

##### Knowledge

Lack of knowledge of guidelines/protocols that prescribe the behaviour has been associated with poor HH compliance [12]. Knowledge of hand rubbing/washing was assessed using demonstration of both hand washing technique (four aspects of the technique were observed e.g., covering the palm and thumbs) [12] and duration (measured as the attendant’s ability to demonstrate the appropriate rubbing/washing for 10 s or more following the Zanzibar infection prevention guidelines). We could not include both variables because of the small sample size in our dataset; we chose to include duration.

Knowledge around avoiding glove recontamination was measured using one item on a 4-point scale (agree a lot, agree a little, disagree a little, disagree a lot). The item asked whether one could pick up germs from touching the delivery surface. It categorized into a binary outcome (agree vs. disagree). We did not use the second knowledge item about glove recontamination, as we were unsure it was correctly interpreted.

##### Candidate Confounders

From the questionnaire, we selected: the type of professional background (e.g., senior nurse, nurse midwife), the time since their last formal training (no training, 1–3 years, 4–7 years, 8 and above), and years served in that specific maternity ward (continuous variable).

### 2.3. Analysis

For the two outcomes, we investigated their association with the same types of modifiable determinants aforementioned with the following exceptions. For avoiding recontamination, we did not consider the availability of single-use drying material, which was not relevant to avoiding recontamination, and knowledge of contamination, which had a limited distribution in our sample. For hand rubbing/washing, we did not consider the time elapsed since donning gloves, which was not relevant, or experiential attitudes because the scale had low internal reliability (Table 1).

All variables were cleaned and checked for inconsistencies. All analyses were carried out at the level of the hand hygiene opportunity. We used cross-tabulations to describe the distribution of variables in our sample overall and by facility. Crude associations between each independent variable and the relevant outcome were calculated using bivariate mixed-effect logistic regression models that accounted for clustering within birth attendants. We then built two separate explanatory mixed-effect logistic regression models (with individual birth attendants as a random effect), one for hand rubbing/washing (Model 1) and one for avoiding glove recontamination (Model 2), to assess which modifiable determinants were independently associated with each of the two outcomes. Model convergence was checked by increasing the number of quadrature points [35].

To construct Model 1 and Model 2, we initially included modifiable determinants postulated above in the following order: (a) contextual variables, (b) knowledge and habit, (c) attitudinal, normative and control beliefs, and (d) other, i.e., received refresher training. We then included all candidate confounders in a stepwise fashion. Potential collinearity between the confounders and the modifiable determinants was assessed by change in the standard error and the mean least square. For variables hypothesized to have a continuous relationship with the outcome, we performed a test for departure from linearity (eventually workload and time since donning gloves were included as categorical variables). Psychological constructs measured as continuous variables were all included as linear terms a priori. Finally, we performed two sensitivity analyses. One assessed the effect on Model 1 of excluding data from the pilot facility; the second assessed the effect on both models of using a different definition of workload (i.e., the number of procedures in the fifteen minutes preceding the index procedure).

All analyses were carried out in STATA v15 SE.

### 2.4. Ethics Approval

The project was approved by the Zanzibar Medical Research and Ethics Committee, the London School of Hygiene and Tropical Medicine Research Ethics Committee, and the Research Ethics Committee at the University of Aberdeen. Consent was obtained from women (patients) either in writing in the antenatal ward prior to observation, or verbally in the labour ward, with written consent obtained before discharge. Women were informed that we would not collect information on them, rather that the person being observed was the birth attendant. Consent to observe the birth attendants was granted by the Ministry of Health and obtained verbally from the birth attendants when data collectors first visited the facility. All healthcare worker information was anonymised. Written consent to administer the questionnaire was obtained from birth attendants directly.

### 2.5. Data Sharing

Anonymised outcome data at the opportunity level is available from https://doi.org/10.17037/DATA.00000778 (“F-Dataset_23-02-2018.xlsx” document). Information on individual level variables is not publicly available because the small sample size may compromise the anonymity of this data. Part of this data can be requested directly from the manuscript authors.

## 3. Results

### 3.1. Descriptive

In total 103 birth attendants were observed and interviewed. Thirty-nine percent of these were nurse midwifes, 24% were orderlies (cleaners), and the rest were a mix of senior nurses, public health nurses B, and others. About half of the sample (51%) was 34 or younger. A quarter of them did not receive any formal training prior to start working, whereas, among those who were trained, their training was on average three years. We observed 779 HH opportunities before aseptic procedures. For 490 of these opportunities, we could also examine glove recontamination. Rubbing/washing compliance was 24.4% (CI: 21.4–27.6, *N* = 779), whilst compliance with avoiding recontamination after donning gloves was 62.0% (CI: 58.0–66.4, *N* = 490). Rubbing/washing compliance did not vary by region (Pemba: 29.6%, *N* = 152; Unguja: 23.1%, *N* = 627) or shift (morning: 24.8%, *N* = 226; afternoon: 26.2%, *N* = 256; night: 22.6% *N* = 297). Similarly, avoiding glove recontamination compliance did not vary by region (Pemba: 61.2%, N = 85; Unguja: 62.2%: *N* = 405) or shift (morning: 66.9%, *N* = 151; afternoon: 60.4%, *N* = 164; night: 59.4%, *N* = 175).

**Figure 1:**
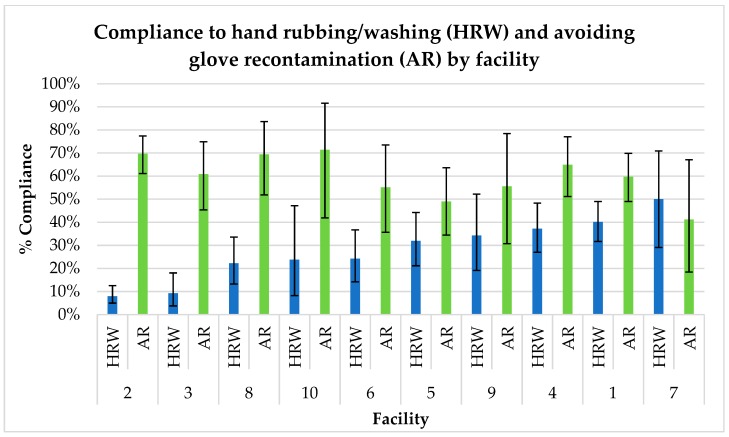
Compliance by facility.

Rubbing/washing compliance varied by facility, whilst avoiding glove recontamination did not (Figure 1). The intracluster correlation coefficient (ICC) for hand rubbing/washing was 12% (CI: 4.8–29.4%) meaning that 12% of the variance lay between facilities, compared to 88% within facilities. The ICC for avoiding glove recontamination was lower, at 0.8% (CI: 0–19.2%). The distribution of certain categorical modifiable determinants was limited in few institutions (see Table 2). For example, availability of single-use drying material and knowledge of hand rubbing/washing showed no variation in three facilities.

This and the following paragraphs describe the distribution of different candidate modifiable determinants by relevant outcome. As Table 3 indicates, compliance with hand rubbing/washing was higher when workload was lower, single-use drying material was present, when birth attendants demonstrated knowledge of the appropriate hand hygiene duration, when they reported hand rubbing/washing to be a habitual behaviour, when they linked poor hand hygiene with negative patient outcomes (instrumental beliefs), when they believed more of their colleagues or managers rubbed/washed hands (descriptive norms), when they reported an environment with more reminders, and when they had received refresher training on hand hygiene in the past 12 months. Self-efficacy was not associated with hand rubbing/washing. From Table 3, only three variables had missing values or values with inconsistent information (each <4%).

Avoiding glove recontamination occurred more frequently when less time elapsed between donning gloves and the index procedure, and when workload was higher (Table 4). The higher the workload, the shorter the time from donning gloves to the index procedure. Counter intuitively, avoiding glove recontamination also occurred more with lower experiential and instrumental attitudes, and when birth attendants had not received refresher training in the last 12 months. Self-efficacy, habit, descriptive norms and reminders were not associated with avoiding glove recontamination. From Table 4, only the variable indicating the presence of the in charge had missing values (1%).

### 3.2. Analytical Models

After adjusting for candidate confounders and all other modifiable determinants in Model 1 (Table 3), higher workload (*p*-value < 0.0001) and the availability of single use drying material (*p*-value = 0.0009) were associated with hand rubbing/washing. When workload was the lowest, the odds of rubbing/washing were 29.4 times higher (CI:12.9–67.0) than when workload was highest. When single use drying material was available, the odds of rubbing/washing were 2.9 times higher (CI: 1.58–5.14) compared to when it was unavailable. We also found weak strength of evidence that having more knowledge (*p*-value = 0.0457) and more reminders (*p*-value = 0.0736) were associated with rubbing/washing. There was no evidence that other candidate modifiable determinants were associated with rubbing/washing.

After adjusting for candidate confounders and the other candidate modifiable determinants in Model 2 (Table 4), the time elapsed since donning gloves until the index procedure was associated with avoiding glove recontamination (*p*-value < 0.0001). When less than a minute elapsed, the odds of avoiding glove recontamination were nearly five times higher compared to when the time elapsed was 3 or more minutes (OR: 4.5, CI: 2.5–8.0). Other candidate modifiable determinants did not appear to be associated with avoiding glove recontamination.

Collinearity was not important in either Model 1 or 2. Our results did not substantially change when we ran the sensitivity analyses (results in Supplementary D).

## 4. Discussion

In our study across the 10 highest volume facilities in Zanzibar, 103 birth attendants were observed using time-and-motion methods and interviewed using a cross-sectional survey. Rubbing/washing compliance was 24.4% (CI: 21.4–27.6) and avoiding glove recontamination was 62.0% (CI: 58.0–66.4). We found that availability of single-use drying material, lower workload, demonstrated knowledge, and an environment with more reminders were determinants associated with more hand rubbing/washing. In contrast, less time elapsed since donning gloves until the procedure was the only factor associated with higher odds of avoiding glove recontamination. This suggests that, at least in the setting of high-volume labour wards in Zanzibar, these two behaviours require different interventions. Moreover, it also suggests that future studies with similar aims should distinguish between hand washing/rubbing and recontamination.

We now consider the possible mechanisms behind the associations we found. The availability of hand hygiene material such as water, soap and drying material to ensure hand hygiene is of course necessary, yet the role of drying material is not often investigated; this is likely to be a more prominent issue in low resource facilities with less research in this area. The importance of single-use drying material was emphasized by Yawson and Hesse in the obstetric/gynaecological units in Ghana [36], since without these, healthcare workers need to air dry which can take several minutes. In environments like labour wards, with unpredictable volumes of patients and needs, spending several minutes drying hands after every hand rubbing/washing opportunity—meaning dozens of times a day in busy wards—is a substantial burden on healthcare workers. Our data collectors reported observing birth attendants adopt tactics to overcome this issue, for example, using the inside of the glove packs, or bringing tissues from home to dry their hands on.

More knowledge [12] or higher workload [11,12,16,17] are associated with hand hygiene compliance in the literature, and we also see these determinants independently associated with hand rubbing/washing in our study. In HH studies, workload is often measured as the number of hand hygiene opportunities preceding the one of interest [15,16]. We believe our measure, based on the number of procedures since the observation start may be a stronger measure as it includes all procedures performed, not just the ones that lead to a HH opportunity. The issue remains that for different opportunities, we had varying lengths of time of observation preceding the opportunity. An observation started with a patient-attendant interaction and hence opportunities closer to the start of observation may have yielded a higher workload as an artefact of our measurement process. We performed sensitivity analyses using a workload variable only based on the previous fifteen minutes of observation, which yielded virtually the same results, giving us confidence that the selected measure of workload did not bias our findings in a significant way. Capturing workload this way was possible because we used time-and-motion methods to observe HH behaviour where all actions were recorded, not just opportunities. A potential key intervention areas is ensuring the availability of gel—very poorly available in this context, but successful at improving hand rubbing/washing and healthcare associated infections in other contexts [37,38,39,40]. Handrub would at once reduce the problems posed by the unavailability of drying material and could save providers’ time as it is quicker to use handrub than to handwash.

Although other studies have investigated the role of perceived normative beliefs on hand hygiene [15,33,41], the specific role of a sanctioning environment has rarely been investigated. Our findings on reminders, indicated that when birth attendants perceived reminders around hand hygiene to be used more frequently in their environment, they were more likely to hand rub/wash. However, reminders may not have been uniformly interpreted by the respondents as sanctioning; some may have interpreted them as a form of supportive supervision or more broadly feedback, which other studies have found to be associated with HH [18]. We could not investigate the role of injunctive norms, because the vast majority of respondents reported very high normative expectations, perhaps due to social desirability. These findings suggest that social influence may play a larger role in Zanzibar maternity units than we have been able to demonstrate in this study.

The less time elapsed since donning gloves until the index aseptic procedure, the higher the odds of avoiding glove recontamination. The change in odds was substantial for each minute added between donning gloves and the procedure. This finding is plausible, in that the longer one keeps gloves on, the greater the chances of touching surfaces that can cause recontamination. This finding should be explored in future studies in the wider healthcare environment. Stressing the importance of donning gloves as close to the point of care as possible may be a useful strategy to prevent glove recontamination. In our dataset, no other variables were associated with avoiding glove recontamination. This is the first attempt to measure determinants of avoiding glove recontamination; it may be that the questions we used to assess the constructs for avoiding glove recontamination were not appropriate. In addition, the use of Likert-like scales is not very common in Zanzibar healthcare workers and may have led to non-differential measurement error; this in turn may have diluted our results towards the null hypothesis; this applies to both our outcomes. Alternatively, our findings may genuinely reflect the lack of strong beliefs or awareness underpinning this behaviour which, compared to hand rubbing/washing, is less emphasized as much in training or supervision in our context. Future studies should investigate the determinants of the two outcomes (hand rubbing/washing and avoiding glove recontamination) separately to assess whether determinants across them differ consistently. In this study we only investigated the determinants of glove recontamination (preceded or not by hand rubbing/washing), but ideally future enquiries could also look into recontamination of the bare hands after hand rubbing/washing (before glove donning).

Residual confounding is a limitation in this study. We were unable to account for the potential confounding effect of each facility itself (which includes infrastructural but also managerial aspects), because the number of facilities (*N* = 10) was too small for a random effect in our model. Additionally, because the distribution of several other key determinants was entirely dependent on the facility, using a fixed effect would have prevented us from investigating key modifiable determinants. In order to investigate further the role of facility, we carried out a detailed spatial and qualitative analysis that is not presented here. A second limitation is that we could also not account for delivery-specific variables, such as the use of delivery sets or obstetric complications, because these were rare exposures. Therefore, we cannot rule out the extent to which these modify the effect of workload. Third, two particular sources of bias may have influenced our findings: Hawthorne effect for the observation tool and social desirability bias for the questionnaire. We tried to minimize the Hawthorne effect, which is common in HH studies—by which we mean the potential alteration of the birth attendants’ behaviour as a consequence of being observed [42,43]—concealing the specific aim of our study from the participants, running a sensitivity analysis removing the one facility that was aware of our study aim, and planning the survey after the observation period. With regards to social desirability in the questionnaire, which cannot be ruled out, the wide distribution of responses observed across the psychological constructs (except for injunctive norms) suggest the effect on average may be modest.

## 5. Conclusions

In conclusion, this multi-centre time-and-motion study combined with a cross-sectional survey, found that availability of single-use drying material, workload, knowledge and an environment with more reminders were associated with hand rubbing/washing. For determinants of avoiding glove recontamination, only time elapse since glove donning was associated. Hence, it seems different determinants underpin the two outcomes investigated—which should be taken into account when developing interventions in this setting. Future studies could further investigate the determinants of avoiding recontamination (both hand and glove), and whether they differ from those driving hand rubbing/washing, in order to develop more appropriate and effective hand hygiene interventions.

## Figures and Tables

**Table 1:** Reliability of psychological constructs measured with Likert-like response scales.

Construct	Number of Items	Cronbach’s alpha
**Outcome 1 (hand rubbing/washing)**		
Instrumental attitudes	5	0.27 *
Experiential attitudes	4	0.31 *
Self-efficacy	4	0.68
Habit	3	0.71
**Outcome 2 (avoiding glove recontamination)**		
Instrumental attitudes	3	0.13 *
Experiential attitudes	2	0.77
Self-efficacy	3	0.76
Habit	3	0.71

* Not used due to low internal reliability.

**Table 2:** Distribution of the categorical modifiable variables by facility for each of our two datasets: hand rubbing/washing dataset (*N* = 779) and avoiding glove recontamination (*N* = 449).

Categorical Modifiable Determinants	Facility % (*n*)									
	1	2	3	4	5	6	7	8	9	10
*N* = 779 *	**Hand rubbing/washing dataset**									
Workload (lowest category)	19.7 (26)	11.9 (24)	21.1 (15)	15.1 (13)	24.6 (17)	24.2 (15)	54.8 (13)	19.4 (14)	25.7 (9)	38.1 (8)
Drying material (not available)	21.2 (28)	99.0 (200)	100 (76)	68.6 (59)	7.3 (5)	100 (62)	100 (24)	98.6 (71)	16 (45.7)	76.2 (16)
Knowledge (duration “>10 s”)	94.7 (125)	34.2 (69)	35.5 (27)	44.2 (38)	36.2 (25)	66.1 (41)	100 (24)	52.8 (38)	54.3 (19)	0 (0)
Instrumental beliefs (mentions hand hygiene)	47.0 (62)	55.5 (112)	36.8 (28)	37.2 (32)	42 (60.8)	80.7 (50)	70.8 (17)	81.9 (59)	31.4 (11)	81.0 (17)
Refresher training (yes)	78.0 (103)	64.4 (130)	382 (29)	24.4 (21)	17.4 (12)	71.0 (44)	41.6 (10)	77.8 (56)	34.3 (12)	71.4 (15)
*N* = 490 **	**Avoiding glove contamination dataset**									
Time since donning gloves (<1 min)	67.4 (62)	59.9 (79)	47.8 (22)	54.4 (31)	49.0 (24)	62.1 (18)	47.1 (8)	69.4 (25)	77.8 (25)	35.7 (5)
Workload (lowest category)	28.3 (26)	15.9 (21)	26.1 (19)	21.1 (12)	34.7 (17)	37.9 (11)	64.7 (11)	33.3 (12)	50.0 (9)	50.0 (7)
Experiential attitudes (always yes a lot)	97.8 (90)	65.9 (87)	82.6 (38)	80.7 (46)	87.8 (43)	27.6 (8)	100 (17)	75.0 (27)	83.3 (15)	42.9 (6)
Instrumental beliefs (mentions hand hygiene)	52.2 (48)	58.3 (77)	36.9 (17)	43.9 (25)	57.1 (28)	72.4 (21)	70.6 (12)	86.1 (31)	38.9 (7)	92.9 (13)
Refresher training (yes)	77.2 (71)	64.4 (85)	39.1 (18)	26.3 (15)	12.2 (6)	65.5 (19)	35.3 (6)	80.6 (29)	38.9 (7)	78.6 (11)

* Sample refers to data available for the hand rubbing/washing outcome. ** Sample refers to data available for avoiding glove recontamination.

**Table 3:** Descriptive characteristics of the sample and adjusted odds ratio (Model 1) for the association between each modifiable determinants and hand rubbing/washing.

Variable Name	Opportunities % (*n*) or Mean (s.d.) *N* = 779	Rubbed/Washed % (*n*) or Mean (s.d.) *N* = 190	Adjusted Odds Ratio * (95% CI) N = 751 **	LRT *p*-Value
**MAIN DETERMINANTS**				
Workload ***				
Highest	20.0 (156)	7.1 (11)	1	<0.0001
High	20.0 (156)	12.8 (20)	1.63 (0.67–3.92)	
Medium	20.0 (156)	25.6 (40)	4.29 (1.90–9.72)	
Low	20.0 (156)	14.7 (23)	2.22 (0.94–5.24)	
Lowest	19.9 (155)	61.9 (96)	29.39 (12.90–67.00)	
**Availability of single use drying material**				
No	71.5 (557)	19.9 (111)	1	0.0009
Yes	26.7 (208)	36.5 (76)	2.85 (1.58–5.14)	
Missing	1.7 (13)	23.1 (3)	-	
Inconsistent info	0.1 (1)	0	-	
**Knowledge (duration)**				
Less than 10 s	47.8 (373)	18.0 (67)	1	0.0457
10 s or more	52.1 (406)	30.3 (123)	1.89 (1.02–3.49)	
**Habit ° (1–10)**	6.12 (2.50)	6.56 (2.40)	1.09 (0.96–1.24)	0.1716
**Instrumental beliefs**				
Does not mention HH	44.8 (349)	21.8 (76)	1	0.8066
Mentions HH	55.2 (430)	26.5 (114)	1.09 (0.55–2.14)	
**Self-efficacy ° (1–10)**	4.9 (3.2)	4.9 (3.1)	0.94 (0.83–1.07)	0.3628
**Descriptive norms (colleagues) ° (1–10)**	5.7 (1.8)	5.9 (1.7)	1.07(0.86–1.32)	0.5309
**Descriptive norms (managers) ° (1–10)**	6.7 (2.3)	6.9 (2.4)	0.95 (0.82–1.11)	0.5646
**Reminders° (0–4)**	2.5 (1.59)	2.9 (1.41)	1.20 (0.98–1.46)	0.0736
**Hand hygiene refresher training in the past 12 months**				
No	44.5 (347)	21.3 (74)	1	0.2390
Yes	55.5 (432)	26.9 (116)	1.43 (0.79–2.59)	
**CONFOUNDERS**				
**Necessary material (water and soap, or gel)**				
No	6.2 (48)	10.4 (5)	1	0.6798
Yes	90.4 (704)	25.1 (177)	1.28 (0.40–412)	
Missing	1.7 (13)	23.1 (3)		
Inconsistent info	1.8 (14)	35.7 (5)		
**Presence of the in charge**				
No	90.9 (708)	23.7 (168)	1	0.3655
Yes	7.5 (58)	32.8 (19)	1.54 (0.61–3.93)	
Missing	1.7 (13)	23.1 (3)	-	
**Professional background**				
Senior Nurse	4.8 (37)	16.2 (6)	1	0.1344
Nurse Midwife	48.7 (379)	23.0 (87)	0.91 (0.21–4.03)	
Public Health Nurse B	10.7 (83)	31.3 (36)	3.17 (0.63–15.87)	
Orderly	9.9 (77)	15.6 (12)	1.89 (0.38–9.50)	
Other nurse or nurse assistant	26.1 (203)	29.1 (59)	1.16 (0.23–5.91)	
**Years since formal training**				
8 and over	21.7(169)	22.5 (38)	1	0.0083
4–7	19.3150)	17.3 (26)	1.50 (0.49–4.58)	
1–3	15.0 (117)	24.8 (29)	4.07 (1.50–11.09)	
No training	9.9 (77)	15.6(12)	- ****	
Years working in this specific maternity °	3.7 (5.2)	3.4 (4.6)	0.99 (0.92–1.06)	0.7102

* Each odds ratio was adjusted for all other variables in the table. ** 28 observations with missing or inconsistent information (variables: in charge, drying material and necessary material) were not included in the model. Model 1 includes overall 182 events. *** Workload was constructed as the number of procedures per minute: 0.3590–1.7647 (highest); 0.2010–0.3589 (high); 0.1129–0.2009 (medium); 0.0502–0.1128 (low); 0–0.0501 (lowest). **** The last category “no training” was omitted because of collinearity with the variable professional background. *No training* in this variable and orderlies in the *professional background* variable were perfectly matched. ° Variables included in the model as linear terms. Reported OR for these variables refers to the effect of one unit increase in the risk factor.

**Table 4:** Descriptive characteristics of the sample, and adjusted odds ratio (Model 2) for the association between each modifiable determinant and avoiding glove recontamination.

Variable Name	Opportunities % (n) or Mean (s.d.) *N* = 490	Clean % (n) or Mean (s.d.) *N* = 304	Adjusted odds * Ratio (95% CI) *N* = 485 **	LRT *p*-Value
**MODIFIABLE DETERMINANTS**				
**Time since donning gloves**				
3 or more minutes	16.9 (83)	39.8 (33)	1	<0.0001
2–3 min	8.0 (39)	35.9 (14)	0.75 (0.33–1.85)	
1–2 min	16.3 (80)	53.8 (43)	1.54 (0.77–3.09)	
Less than a minute	58.8 (288)	74.3 (214)	4.49 (2.51–8.04)	
Workload ***				
Lowest	28.2 (138)	55.1 (76)	1	0.4694
Low	227 (111)	60.4 (67)	1.29 (0.72–2.34)	Test for trend= 0.0641
Medium	19.4 (95)	65.3 (62)	1.42 (0.75–2.69)	
High	17.8 (87)	65.5 (57)	1.64 (0.84–3.23)	
Highest	12.0 (59)	71.2 (42)	1.87 (0.87–4.04)	
**Habit ° (1–10)**	6.6 (2.5)	6.6 (2.9)	0.99 (0.89–1.10)	0.8005
**Experiential attitudes**				
Mixed responses	23.1 (113)	65.5 (74)	1	0.6505
Always responded yes a lot	76.9 (377)	61.0 (230)	1.18 (0.57–2.45)	
**Instrumental beliefs**				
Does not mention HH	56.9 (279)	67.4 (188)	1	0.1670
Mentions HH	43.1 (211)	55.0 (116)	1.52 (0.83–2.78)	
**Self-efficacy ° (1–10)**	4.9 (3.2)	4.9 (3.1)	1.02 (0.94–1.11)	0.6993
**Descriptive norms (colleagues) ° (1–10)**	6.0 (2.0)	5.9 (2.1)	1.06 (0.86–1.23)	0.5551
**Descriptive norms (managers)° (1–10)**	6.9(2.4)	6.8 (2.5)	0.90 (0.76–1.05)	0.1731
**Reminders° (0–4)**	2.6 (1.6)	2.6 (1.6)	1.03 (0.87–1.23)	0.7831
**Hand hygiene refresher training in the past 12 months**				
No	54.5 (267)	64.0 (171)	1	0.6245
Yes	45.5 (223)	59.6 (133)	1.16 (0.65–2.05)	
**CONFOUNDERS**				
**Knowledge (touching delivery surface one can pick up germs)**				
Agree	95.7 (469)	61.2 (287)	1	0.8171
Disagree	4.3 (21)	81.0 (17)	1.22 (0.23–6.53)	
**Presence of the in-charge**				
No	90.8 (445)	62.0 (276)	1	0.4205
Yes	8.2 (40)	67.5 (27)	1.44 (0.59–3.56)	
Missing	1.0 (5)	20.0 (1)		
**Professional background**				
Senior Nurse	3.9 (19)	68.4 (13)	1	0.2252
Nurse Midwife	52.7 (258)	59.7 (154)	0.39 (0.10–1.68)	
Public Health Nurse B	9.8 (48)	58.3 (28)	0.71 (0.15–3.38)	
Orderly	6.7 (33)	51.5 (17)	0.35 (0.08–1.68)	
Other nurse or nurse assistant	26.9 (132)	69.7 (92)	0.67 (0.13–3.39)	
**Years since formal training**				
8 and over	20.0 (98)	64.3 (63)	1	0.3938
4–7	22.2 (109)	66.1 (72)	0.82 (0.32–2.10)	
1–3	51.0 (250)	60.8 (152)	1.40 (0.53–3.74)	
No training	6.7 (33)	51.5 (17)	- ****	
**Years working in this specific maternity °**	3.5 (4.2)	3.24 (3.6)	0.94 (0.87–1.02)	0.1082

* Each odds ratio was adjusted for all the other variables in the table. ** 5 observations with missing or inconsistent information (variables: in charge) were not included from model. Model 2 includes overall 303 events. *** Workload was constructed as the number of procedures per minute: 0.3590–1.7647 (highest); 0.2010–0.3589 (high); 0.1129–0.2009 (medium); 0.0502–0.1128 (low); 0–0.0501 (Lowest). **** The last category “no training” was omitted because of collinearity with the variable professional background. *No training* in this variable and orderlies in the *professional background* variable were perfectly matching. ° Variables included in the model as linear terms. Reported OR for these variables refers to one unit increase in the risk factor.

## Supplementary Materials

The following are available online at https://www.mdpi.com/1660-4601/17/4/1438/s1. Supplementary A: Sample size scenarios, Supplementary B: Questionnaire, Supplementary C: selection of modifiable determinants, Supplementary D: Sensitivity analyses. Anonymised outcome data at the opportunity level is available from https://doi.org/10.17037/DATA.00000778. Information on individual level variables is not publicly available because the small sample size may compromise the anonymity of this data. Part of this data can be requested directly from the manuscript authors.
