# Supplementary material for: Behavioural Determinants of Hand Washing and Glove Recontamination before Aseptic Procedures at Birth: A Time-and-Motion Study and Survey in Zanzibar Labour Wards
Source: Int J Environ Res Public Health. 2020 Feb 24;17(4):1438. doi: 10.3390/ijerph17041438 (PMC7068290; doi:10.3390/ijerph17041438)
Supplement: Supplementary file 1 [file ijerph-17-01438-s001.pdf]

International Journal of
Environmental Research and Public Health

MDPI

Supplementary A – Sample size scenarios

Sample size scenarios were calculated in EpiInfo v7 using the Fleiss' formula with continuity correction. Cells BELOW the bold line fit within our sample size.

Number of opportunities for 5:1 unexposed to exposed ratio.

5:1	Effect size							
% of outcome in Unexposed	1.1	1.3	1.5	1.8	2	2.5	3	5
3%	191567	23213	9036	3920	2672	1365	864	309
5%	112504	13617	5294	2291	1559	794	501	176
10%	59820	6420	2487	1071	725	365	227	75
15%	33450	4020	1552	663	447	222	136	41
40%	8745	1022	381	153	98			

* Power at 80% and confidence intervals at 95%.

Number of opportunities for 2:1 unexposed to exposed ratio.

2:1	Effect size							
% of outcome in Unexposed	1.1	1.3	1.5	1.8	2	2.5	3	5
3%	120483	14771	5810	2555	1755	912	587	216
5%	70758	8660	3402	1493	1023	530	339	123
10%	33450	4079	1595	695	474	243	153	53
15%	21018	2552	993	429	291	147	92	29
40%	5478	642	240	96	62			

*Power at 80% and confidence intervals at 95%.

Number of opportunities for 1:1 unexposed to exposed ratio.

1:1	Effect size							
% of outcome in Unexposed	1.1	1.3	1.5	1.8	2	2.5	3	5
3%	107756	13350	5298	2356	1628	856	556	210
5%	63364	7824	3100	1374	938	496	320	118
10%	29900	4190	1452	144	438	226	155	50
15%	18780	3002	902	110	268	136	84	26
40%	4878	574	214	86	56			

*Power at 80% and confidence intervals at 95%.

Supplementary B – Questionnaire

Birth Attendant Questionnaire

Introduction

I am ______________________________ from ______________________

Purpose of the study

- To assess how well hand washing is done in maternity units and at home.
- To understand when and why health care staff wash their hands when helping during child birth.
- To design a programme to improve hand washing by health care staff.

This project is in partnership with the Ministry of Health and your co-operation is very important to ensure its success and ultimately improve infection prevention practices in Zanzibar. All birth attendants across 10 high volume hospitals in Unguja and Pemba will be asked to participate.

Conditions for participation
We would like you to take part in one interview. We will ask you questions about your experience of supervising or working in a maternity unit or caring for your newborn baby. The interview will take 30 to 45 minutes.

Risk or Discomfort
We may ask questions that you feel shy to answer or you do not want to answer. If this happens, you can refuse to answer or you can end the interview. You may find taking part in the interview is tiring. To minimize this, we will use a short structured questionnaire

Cost/Compensation
Taking part in this study will not cost you anything. You will not be paid but you will be compensated for your time.

Contact person for further questions or complaints
You can ask the interviewer any questions and raise any concerns. If they cannot help they will pass the question onto a senior member of the team. You may contact directly: Dr Said Ali, Director Public Health Laboratory, P.O.BOX 122 Wawi, Chake Chake, Pemba. Tele/Fax +255 24 2452003

Confidentiality

All information will be kept strictly confidential. Your name and any identifying information will be removed from publications so it will not be possible to link the responses to any particular person or setting. If you are not comfortable with any findings being shared, we will not publish them in order to protect your identity. Identifiable information will not be shared with your colleagues of facility managers

Voluntary participation
Taking part in the interview is voluntary and you are free to withdraw at any time. If you decide that you do not want to take part we will respect your decision. There will be no complaint or punishment.
Permission to continue
"Do you have any questions for me?"

If informant has any questions, record questions and your response here:

I, ____________________ (name of the respondent) have read and understood this text, understand what is expected of me and all my questions have been answered. I understand that I can withdraw at any time without giving any reason and this will not affect my work or any health services entitled to me. I freely accept to participate in this study.

______________________ ______________________ **Date**: __________
Respondent's name *Signature (thumbprint)*

______________________ ______________________ **Date**: __________
Interviewer's name *Signature*

Now we are going to start the interview. Feel free to ask any clarification and questions. If you do not understand a question, do not hesitate to ask.

	**Respondent Information**	
R1	Respondent code (ID)	☐☐
R2A	Interviewer code (ID)	☐☐
R2B	Facility code (ID)	☐☐
R3	Date<br>**Write the date in NUMBERS for day month year (DDMMYYYY)**	DATE ☐☐ MONTH ☐☐ YEAR ☐☐☐☐
R4	Time<br>**Write the start time using the 24 hour clock format e.g., 0815**	☐☐☐☐
R5A	What is your title?	(1) Senior Nurse<br>(2) Nurse Midwife<br>(3) Public Health Nurse B (PHNB)<br>(4) Maternal and Child Health Aid (MCHA)<br>(5) Orderly<br>(6) Clinical officer<br>(7) Gynaecologist<br>(8) Assistant Medical Officer<br>(88) Other (Please specify)
R5B	Are you the maternity in charge?	(1) Yes<br>(2) No
R6	How long is your service at this maternity ward (yrs/months)?:	**Years** ☐☐ **Months** ☐☐
R7	How many years of service since completing your latest formal training for this position?	**If no training, put 99** ☐☐
R8	How old are you?	☐☐
R9	To become a birth attendant, how many years in total did you spend in formal training?	**If no training, put 99** ☐☐
	**Household characteristic**	
R10A	How many household members are 18-years old or younger?	☐☐
R10B	Are all household members aged 6 to 18 currently students?	(1) Yes<br>(2) No<br>(3) No members ages 6 to 18
R10C	What is the main building material used for the walls of the main building of your house?	(1) Baked bricks<br>(2) Poles and mud, grass, sun-dried bricks, or other<br>(3) Stones, cement bricks, or timber
R10D	What is the main fuel used for cooking?	(1) Firewood, solar, gas (biogas), or wood residuals,

		(2) Charcoal, paraffin, gas (industrial), electricity, generator/private source, or other
R10E	Does your household have any televisions?	(1) Yes
		(2) No
R10F	Does your household have any radios, cassette/tape recorders, or hi-fi systems?	(1) Yes
		(2) No
R10G	Does your household have any lanterns?	(1) Yes
		(2) No
R10H	Does your household have any tables?	(1) Yes
		(2) No
R10I	In the last 12 months, has your nuclear family cultivated any crops?<br>Does your nuclear family own cattle?	(1) No crops, and no cattle
		(2) No crops, and yes cattle
		(3) Yes crops, but no cattle
		(4) Yes crops, and yes cattle
R10J	What is the main building material used for the roof of the main building?	(1) Leaves (makuti), or other
		(2) Iron sheets (tin)/asbestos, concrete, tiles
**Training and supervision**		
F1	Think about the last two weeks - how much time during a day does the maternity in charge spend in the delivery room?	(1) Less than half an hour
		(2) Between 30 minutes and 2 hours
		(3) More
F2	If there is an emergency during labour/delivery, who do you usually contact?	(1) The maternity in-charge
		(2) Another colleague or birth attendant
		(3) Doctor on call
		(4) Other (please specify)
3	Did you receive any refresher training including hand hygiene in the past 12 months?	(1) Yes
		(2) No
F4	Did you attend any supportive supervision session in the past three months?	(1) Yes
		(2) No
I1B	A mother brings her 1 week old baby to the pediatric ward with a high fever. You examine the baby and discover the umbilical cord is infected – it is red and discharging pus. List all the possible causes that might have caused the infection<br><br>*Instructions: Encourage the respondent to identify as many sources as possible and to be* ***as specific as possible***	(1) Mentions hand hygiene of birth attendant
		2) Does not mention hand hygiene of birth attendant

Please remember that there are no good or bad answers, just the answers that best describe how you think and feel about these issues. The topic will be hand hygiene during delivery. When I ask you about hand hygiene I mean hand washing with water <u>and soap</u> or handrubbing with the appropriate duration and technique, drying.

**Experiential attitudes**				
		**No**	**Yes – A little**	**Yes – A lot**
		(1)	(2)	(3)
	**I am going to ask you some questions. Let’s start with one easy to practice:**			
A1	Do you feel **hungry** before lunch?			

A2	Do your hands feel **dry** when you wash your hands repeatedly? *Note for interviewers: repeatedly means you have washed your hands 40 times across 10 women in labour*
A3	Do you feel like a **good nurse** when you wash your hands before a delivery?
A4	Do you feel **anxious** (wasi wasi) when you do **not** wash your hands before a delivery?
A5	Do you feel **uncomfortable** when you put on gloves with wet hands?
A6	**In a situation when you are working alone and there are multiple women to assist.** Do you feel **like you are wasting time/have insufficient time (kupoteza muda wako)** when you wash yours hands before a delivery?

**Instrumental attitudes**					
I1A	Among newborns born in healthcare facilities, some newborns develop an infection within 1 week from birth. Where do you think infections com from?	Home (1)	Facility (2)	Similar for both (3)	
	**I am going to read you some statements, and I will ask you if you agree with them or not**	Agree a lot	Agree a little	Disagree a little	Disagree a lot
		(1)	(2)	(3)	(4)
I2	Before a delivery, if a nurse forgets to wash her hands, **she will get an infection**. Do you agree or disagree? A little or a lot?				
I3	Before a delivery, washing your hands will prevent the **newborn** from developing a serious infection. Do you agree or disagree? A little or a lot?				
I4	A newborn does not develop and infection if the birth attendant wears gloves but forgets to hand wash. Do you agree or disagree? A little or a lot?				
I5	You are wearing **two layers** of gloves at the same time. After having assisted a delivery, **you have taken the top layer off** and you immediately perform another delivery. In this situation, your hands will cause the newborn an infection. Do you agree or disagree? A little or a lot?				

**Perceived control/Self-efficacy**				
	**In some situations, nurses find it difficult to wash their hands with soap before a delivery. I am going to ask you questions about your ability to perform hand washing in difficult situations**			
		Ve[illegible]	A[illegible]	N[illegible]
		(1)	(2)	(3)
C1	How sure are you that you will (can) wash hands before **every** delivery when **water is not flowing** from the sink?			
C2	How sure are you that you will (can) wash hands before **every** delivery when **there is no disposable drying material**?			
C3	How sure are you that you will (can) wash hands before **every** delivery when **there is no birth attendant available to assist you** during delivery?			
C4	How sure are you that you will (can) wash hands before **every** delivery **when a mother is rushed** into the ward just before delivery, and you can see the **newborn's head on the perineum**?			

**Social Influence and social norms**				
		**No**	**Yes usually**	**Yes always**
		(1)	(2)	(3)

N1A	Do you think **you should** (napasso) wash your hands before a delivery?			
N2A	*[We recently asked 10 birth attendants in Zanzibar that previous question]*<br><br>Out of these10, how many birth attendants thought **they should always** wash hands before a delivery?	☐☐		
N3A	Think about birth attendants in Zanzibar. Out of 10, how many do you think do **always wash** hands before a delivery?	☐☐		
N3B	Think about **in-charge(s)** in Zanzibar. Out of 10, how many do you think do **always wash** hands before a delivery?	☐☐		
N1B	In a situation when you are working alone with multiple women to assist.<br>Do you think **it is OK to not** wash your hands before a delivery in this circumstance?	Ne[illegible]<br>(1)	Not usua[illegible]<br>(2)	OK to<br>(3)
N2B	*[We recently asked 10 birth attendants in Zanzibar that previous question]*<br><br>Out of these10, how many birth attendants thought **it is never OK to** wash hands when they are working alone and assisting multiple women?	☐☐		
N4A	In the past month, have **you** reminded anyone to wash hands?	(1) Yes<br>(2) No<br>(3) Don't remember		
N4B	In the past month, has **anyone** reminded you to wash hands?	(1) Yes<br>(2) No<br>(3) Don't remember		
N4C	In the past month In the past month have you seen or heard that **the ward manager** reminded anyone to wash hands?	(1) Yes<br>(2) No<br>(3) Don't remember		
N4D	In the past month has **any of your colleagues** reminded anyone to wash hands?	(1) Yes<br>(2) No<br>(3) Don't remember		

Habit				
**Sometimes at work things come automatically and sometimes you have to remind yourself**		**No**	**Yes, usually**	**Yes, always**
		(1)	(2)	(3)
H1	Do you wash your hands before a delivery **without thinking**?			
H2	Do you start hand washing before a delivery **without even realising** you are doing it?			
H3A	Do you ever have to **remind yourself** to hand wash before a delivery?			
	**"Sometimes if people do things again and again, it becomes automatic and we don't need to think about it anymore. For example, when I first start fasting, I needed to consciously remember to not eat, but after a few days, I did it automatically without even thinking. Do you understand?"**			
H3B	In a situation when you are working alone with multiple women to assist.<br>Do you **automatically** wash hands before a delivery?			

**Intention**				
	**"Remember, there are no "good" or "bad" answers - we would like you to answer honestly. You personal answers will not be shared with anyone"**	No	Yes, usually	Yes, always
		(1)	(2)	(3)
S1	Do you intend to wash your hands before every delivery?			
S2	Do you expect to wash your hands before every delivery when you are working alone?			
S3	Do you plan to wash your hands before a delivery when the birth is very fast (woman rushed into the labour room and the head is on perineum)?			

Knowledge					
	**I am going to read you some statements, and I will ask you if you agree with them or not**	Agree a lot	Agree a little	Disagree a little	Disagree a lot
		(1)	(2)	(3)	(4)
K1	Before a delivery, rubbing hands with hand gel is **not as effective** as water and soap at disinfecting. Do you agree or disagree? A little or a lot?				
K2	When yours hands touch very briefly the **trolley**, they **cannot** pick up germs. Do you agree or disagree? A little or a lot?				
K3	When your hands touch very briefly the **Macintosh or kanga**, they **can** pick up germs. Do you agree or disagree? A little or a lot?				
K4	Hand gel dries off in	(1) Less than 10 seconds<br>(2) between 10 and 20 seconds<br>(3) between 20 and 60 seconds			
K5	Please demonstrate the appropriate hand washing technique and duration	Record if she shows:<br>☐ Behind fingers<br>☐ Thumbs<br>☐ Interlocking fingers (nail-fingers in Swahili)<br>☐ Palm			
K6		Duration in seconds:<br>1) Less than 10<br>2) 10+			
K7	Which one takes longer: hand washing or hand rubbing?	(1) Hand washing<br>(2) Hand rubbing<br>(3) Don't know			

Take a couple minutes break

Sometimes birth attendants touch unsterile objects like the register or the phone before a delivery. I want to ask you some questions about this.

Experiential attitudes				
		No	Yes - A little	Yes - A lot
		(1)	(2)	(3)
A7	When you briefly touch the **register, pen, or phone** after putting on gloves, do you feel your hands are too dirty to conduct a delivery?			
A8	In a situation when you are working alone with multiple women to assist.<br>Do you feel **anxious** when you briefly touch a **register, pen, or phone** after putting on gloves to conduct a delivery?			
A9	Do you feel **proud of completing things** (kufanikisha) when you avoid touching unsterile objects before conducting a delivery?			

Instrumental attitudes					
	**I am going to read you some statements, and I will ask you if you agree with them or not**	Agree a lot	Agree a little	Disagree a little	Disagree a lot

		(1)	(2)	(3)	(4)
I6	Touching a **register, pen, or phone** just before cord-cutting will cause an infection to the newborn. Do you agree or disagree? A little or a lot?				
I7	Avoiding touching a **kanga or Macintosh** before touching a woman's vagina will prevent her a serious infection. Do you agree or disagree? A little or a lot?				
I8	My hands are gloved and I am ready to perform a delivery. If I wash my hands again every time I touch a Macintosh or kanga I do not get any work done.				
I9	If I touch a **pen or phone or register** before touching a woman's vagina, I will get reprimanded.				

**Perceived control/Self-efficacy**				
		Ve [illegible]	A [illegible]	N [illegible]
	**BEFORE A DELIVERY**	(1)	(2)	(3)
C5	How sure are you that you will (can) avoid touching unsterile objects **when the delivery equipment is in various places**?			
C6	How sure are you that you will (can) avoid touching unsterile objects **when you are working alone**?			
C7	How sure are you that you will (can) avoid touching unsterile objects **when you are responsible for assisting multiple women at second stage labour**?			

**Social Influence and social norms**				
		**Ne ve**	**N ot**	**O K**
		(1)	(2)	(3)
N5A	Do you think you **can** touch unsterile objects just after putting on gloves for a delivery?			
N6A	*We recently asked 10 birth attendants in Zanzibar the previous question.*<br><br>Out of these10, how many birth attendants thought **they should never** touch unsterile objects after putting on gloves for a delivery?	☐☐		
N7A	Think about birth attendants in Zanzibar. Out of 10, how many **never touch** unsterile objects after putting on gloves for a delivery?	☐☐		
N7B	Think about **in-charge(s)** in Zanzibar. Out of 10, how many **never touch** unsterile objects after putting on gloves for a delivery?	☐☐		
N5B	**In a situation when you are working alone with multiple women to assist.**<br>Do you think **it is OK** to touch unsterile objects just after putting on gloves for a delivery in this circumstance?	**Ne ve**<br>(1)	**N ot**<br>(2)	**O K**<br>(3)
N6B	*We recently asked 10 birth attendants in Zanzibar the previous question.*<br><br>Out of these10, how many birth attendants thought **they should never** touch unsterile objects after putting on gloves for a delivery, when they are **working alone and they are assisting multiple women**?	☐☐		
N8A	In the past month have **you** reminded anyone to not touch unsterile objects after putting gloves on for a delivery?	(1) Yes<br>(2) No<br>(3) Don't remember		
N8B	In the past month has **anyone** reminded you to not touch unsterile objects after you put your gloves on before a delivery?	(1) Yes<br>(2) No<br>(3) Don't remember		
N9A	In the past month have you seen or heard that **the maternity in-charge** reminded anyone not to touch unsterile objects?	(1) Yes<br>(2) No<br>(3) Don't remember		
N9B	In the past month has **any of your colleagues reminded** anyone to not touching unsterile objects?	(1) Yes<br>(2) No<br>(3) Don't remember		

**Habit**				
		No	Yes, usually (most of the times)	Yes, always (all the times)
**Sometimes at work things come automatically and sometimes you have to remind yourself**		(1)	(2)	(3)
H4	Do you avoid touching unsterile objects before a delivery **without thinking**?			
H5	Do you avoid touching unsterile objects before a delivery **without realising** it?			

H6	Do you need to **remind yourself** to avoid touching unsterile objects before a delivery?			
	**"Sometimes if people do things again and again, it becomes automatic and we don't need to think about it anymore. For example, when I first started fasting, I needed to consciously remember to not eat, but after a few days, I did it automatically without even thinking. Do you understand?"**			
H7	**In a situation when you are working alone with multiple women to assist.** Do you **automatically** avoid touching unsterile objects before a delivery?			

**Intention**				
**"Remember, there are no "good" or "bad" answers - we would like you to answer honestly. You personal answers will not be shared with anyone"**		No	Yes, usually	Yes, always
		(1)	(2)	(3)
S4	Do you intend to avoid touching unsterile objects before a delivery?			
S5	Do you expect to avoid touching unsterile objects before a delivery when you are working alone?			
S6	Do you plan to avoid touching unsterile objects before a delivery when the birth is very fast (woman rushed into the labour ward and head is on perineum)?			

**THE END**
**Do you have any comments or questions after completing this questionnaire?**

Thank you for your participation in this study and for completing this questionnaire!

**QUESTIONS FOR INTERVIEWER**		
Q1	Is there any reason for you to believe that the respondent did not understand the answer categories? [If yes, explain what caused you to think this]	(1) Yes Why?<br>(2) No

Supplementary C – selection of modifiable determinants

Table 1 – List of modifiable exposures

Variable	Questionnaire reference/Observation tool	How it was measured
**Both outcomes**		
1. Attended supportive supervision in the last 3 months	Questionnaire – F4	Categorical response options
2. Hand hygiene refresher training in the past 12 months	Questionnaire – F3	Categorical response options

3. Instrumental beliefs (scenario)	Questionnaire – I1B	Binary response option
4. Delivery equipment type used	Observation – collected at the time of delivery	Categorical response options
5. Workload (number of procedures per minute)	Observation – procedures collected throughout observation as they happen	Composite variable described in manuscript methods section
6. Presence of the in charge	Observation – collected at the beginning of every observation session; updates after it changes	Categorical response options
**Outcome 1**		
1. Availability of single-use drying material	Observation – collected at the beginning of every observation session; updates after it changes	Categorical response options
2. Availability of gloves	Observation – collected at the beginning of every observation session; updates after it changes	Categorical response options
3. Necessary hand hygiene equipment	Observation – collected at the beginning of every observation session; updates after it changes	Composite variable made of three individual items with categorical response options: availability of water & soap, or gel. Described in manuscript methods section
4. Knowledge – technique demonstration	Questionnaire – K5	Number of all technique items when demonstrating handwashing
5. Knowledge – duration demonstration	Questionnaire – K6	Binary response option
6. Habit	Questionnaire – H1, H2, H3A, H3B	Likert scale response
7. Self-efficacy	Questionnaire – C1, C2, C3, C4	Likert scale response
8. Experiential attitudes	Questionnaire – A2, A3, A4, A5, A6	Likert scale response
9. Instrumental attitudes	Questionnaire – I2, I3, I4, I5	Likert scale response
10. Injunctive norms	Questionnaire – N1A, N2A, N1B, N2B	N1A – Likert scale<br>N2A – Response out of 10
11. Descriptive norms	Questionnaire – N3A, N3B	Response out of 10
12. Reminders	Questionnaire – N4A, N3B, N4C, N4D	Composite variable made of N4A, N3B, N4C, N4D<br>Individual items have categorical response options. Described in manuscript methods section
**Outcome 2**		
1. Time since donning gloves	Observation – automatically logged time once actions are recorded	Continuous variable described in manuscript methods section
2. Knowledge	Questionnaire – K2, K3	Likert scale response
3. Habit	Questionnaire – H4, H5, H6, H7	Likert scale response
4. Self-efficacy	Questionnaire – C5, C6, C7	Likert scale response
5. Experiential attitudes	Questionnaire – A7, A8, A9	Likert scale response
6. Instrumental attitudes	Questionnaire – I6, I7, I8 and I9	Likert scale response
7. Injunctive norms	Questionnaire – N5A, N6A, N5B, N6B	N5A– Likert scale<br>N6A– Response out of 10
8. Descriptive norms	Questionnaire – N7A, N7B	Out of 10 response
9. Reminders	Questionnaire – N8A, N8B, N9A, N9B	Composite variable made of N8A, N8B, N9A, N9B

	Individual items have categorical response options.

Table 2 - Excluded variables and reason for exclusion

Variable	Reason for exclusion
**Both outcomes**	
Attended supportive supervision in the last 3 months	Limited distribution in sample 93.2% (n=726) did not attend supervision
Delivery equipment type used	Only related to delivery variable. Insufficient deliveries (N=170) in the dataset
Presence of in-charge	Limited distribution in sample; in 90.8% (n=708) in-charge was not present.
**Outcome 1**	
Availability of gloves	Limited distribution in sample; 96.5% (752) had gloves
Necessary hand hygiene equipment	Limited distribution in sample; 90.4% (704) had the necessary equipment
Knowledge – technique demonstration	Knowledge – duration was chosen instead.
Injunctive norms	N1A - Limited distribution in sample; 85% responded *always*.
	N2A - Limited distribution in sample; 85% responded *10/10 colleagues*
**Outcome 2**	
Knowledge	K2 – Concerns about the interpretation of this question. K3 seemed a better choice.
	K3 – Limited distribution in sample; 95.7% agreed with statement
Injunctive norms	N5A - Limited distribution in sample; 93% responded *never*
	N6A - Limited distribution in sample - 58% responded *10/10 colleagues*

Table 3 – Psychological constructs construction; excluded variables based on internal reliability or direction of association

Construct	Reason for exclusion of particular item	Scale Cronbach's alpha	If scale could not be constructed, what was the reason?
**Outcome 1**			
Self-efficacy	No items excluded	0.68	Not applicable
Experiential attitudes	A3 had poor spread	0.31	Low internal reliability, possibly due to formulation of items. Dropping individual items would have lowered Alpha even further. Although items A2, A5, A6 intended as reverse-scored, lack of inverse correlation indicated that A5 had not been understood as reverse-scored.
Instrumental attitudes		0.27	Low internal reliability. I4 only was intended to be scored in the opposite direction from the other three items, but in fact I3 scores also had inverse correlations with I2 and I5. Dropping I3 would not increase overall Alpha sufficiently (only achieving 0.31).
Habit	Excluded H3A, as its intended reverse scoring did not work.	0.71	Not applicable
**Outcome 2**			

Experiential attitudes	A9 was excluded as it had poor spread. Its removal increased the alpha from an initial 0.58.	0.77 for scale with A7 and A8 alone.	Not applicable
Instrumental attitudes	Not applicable	0.13	Low internal reliability. Removing any of the items would not have improved the result substantially.
Self-efficacy	Not applicable	0.76	Not applicable
Habit	Removed item H6, as the intended reverse scoring did not work.	0.71	Not applicable

Supplementary D – Sensitivity analyses

Table 1 – Sensitivity 1 – Remove pilot facility from Model 1

**Variable name**	**Adjusted odds ratio* (95% CI) N=727****
**Workload*****	
Highest	1
High	1.42 (0.58-3.48)
Medium	4.20 (1.85-9.52)
Low	1.94 (0.80-4.67)
Lowest	31.10(13.44-71.99)
**Availability of single use drying material**	
No	1
Yes	3.19 (1.76-5.78)
**Knowledge (duration)**	
Less than 10 seconds	1
10 seconds or more	1.71(0.90-3.25)
**Habit°**	1.09 (0.96-1.24)
**Instrumental beliefs**	
Does not mention HH	1
Mentions HH	1.26 (0.64-2.48)
**Perceived control°**	0.94 (0.83-1.07)
**Descriptive norms (colleagues)°**	1.11(0.89-1.39)
**Descriptive norms (managers)°**	0.94 (0.80-1.10)
**Reminders°**	1.18 (0.97-1.45)
**Hand hygiene refresher training in the past 12 months**	
No	1
Yes	1.72 (0.93-3.18)

*Each odds ratio is adjusted for all other variables in the table

**Compared to Model 1 in the manuscript, 24 observations were not included from model because they belong to the pilot facility which has 12 events.

***Workload was constructed as the number of procedures per minute: 0.3590-1.7647 (highest); 0.2010-0.3589 (high); 0.1129-0.2009 (medium); 0.0502-0.1128 (low); 0-0.0501 (lowest)

°Variables included in the model as linear terms. Reported OR for these variables refers to one unit increase.

Table 2 – Sensitivity Model 1 and 2 with a different measure of workload (i.e., the number of procedures in the fifteen minutes proceeding the index procedure)

Hand rubbing/washing (Model 1)	
**Variable name**	**Adjusted odds ratio* (95% CI) N=629****
**Workload (procedures number in 15 minutes preceding the index procedure)**	
2 or more	1
1	9.00 (3.18-25.47)
0	21.7 (8.31-56.44)
**Availability of single use drying material**	
No	1
Yes	1.77 (0.93-0.23)
**Knowledge (duration)**	
Less than 10 seconds	1
10 seconds or more	2.17 (1.15-4.12)
**Habit°**	1.08 (0.94-1.23)
**Instrumental beliefs**	
Does not mention HH	1
Mentions HH	1.23 (0.62-2.59)
**Perceived control°**	0.95 (0.83-1.09)
**Descriptive norms (colleagues)°**	1.17(0.93-1.45)
**Descriptive norms (managers)°**	0.91 (0.76-1.06)
**Reminders°**	1.17 (0.96-1.42)
**Hand hygiene refresher training in the past 12 months**	
No	1
Yes	1.79 (0.96-3.37)

*Each odds ratio is adjusted for all other variables in the table

**Compared to Model 1 in the manuscript, 122 observations were not included in the model because they did not have a period of 15 minutes preceding them.

°Variables included in the model as linear terms. Reported OR for these variables refers to one unit increase.

Avoiding glove recontamination (Model 2)	
**Variable name**	**Adjusted odds* ratio (95% CI) N=386****
**Time since donning gloves**	
3 or more minutes	1
2-3 minutes	0.76 (0.30-1.90)
1-2 minutes	1.30 (0.60-2.80)
Less than a minute	4.84 (2.52-9.29)
**Workload (procedures number in 15 minutes preceding the index procedure)**	
2 or more	1
1	1.70 (0.72-4.04)
0	0.95 (0.45-2.02)
**Habit°**	1.02 (0.91-1.15)
**Experiential attitudes**	
Mixed responses	1
Always responded yes a lot	0.99 (0.46-2.16)
**Instrumental beliefs**	
Does not mention HH	1
Mentions HH	1.18 (0.61-2.28)
**Perceived control°**	0.97 (0.86-1.10)

**Descriptive norms (colleagues)°**	1.01 (0.82-1.23)
**Descriptive norms (managers)°**	0.88 (0.74-1.05)
**Reminders°**	0.99 (0.82-1.19)
**Hand hygiene refresher training in the past 12 months**	
No	1
Yes	1.08 (0.57-2.02)

*Each odds ratio is adjusted for all other variables in the table

**Compared to Model 2 in the manuscript, 99 observations were not included here because they did not have a period 15 minutes preceding them.

°Variables included in the model as linear terms. Reported OR for these variables refers to one unit increase.
